# A case report of postsplenectomy reactive thrombocytosis leading to an acute myocardial infarction in a previously healthy adult without hematologic comorbidities

**DOI:** 10.1097/MD.0000000000042459

**Published:** 2025-05-09

**Authors:** Chulhyo Jeon, Kiyoung Sung, Jinbeom Cho

**Affiliations:** a Department of Surgery, Uijeongbu St. Mary’s Hospital, College of Medicine, The Catholic University of Korea, Seoul, Republic of Korea; b Department of Surgery, Bucheon St. Mary’s Hospital, College of Medicine, The Catholic University of Korea, Seoul, Republic of Korea.

**Keywords:** complications, splenectomy, thrombocytosis

## Abstract

**Rationale:**

Reactive thrombocytosis frequently occurs after splenectomy. While splenectomy-induced reactive thrombocytosis (SI-RT) might be linked to thromboembolic risks, the causative relationship remains unclear.

**Patient concerns:**

A 72-year-old male with hypertension underwent elective splenectomy for a large splenic hematoma. Postoperative recovery was uneventful until day 11, when he developed sudden epigastric pain and vomiting.

**Diagnoses:**

Laboratory findings revealed significant thrombocytosis (platelet count: 972 K/μL) and elevated troponin levels, with an electrocardiogram confirming an acute ST-segment elevation myocardial infarction. Coronary angiography identified triple-vessel disease with total occlusion of the proximal left anterior descending artery.

**Interventions:**

The patient was successfully treated with a drug-eluting stent.

**Outcomes:**

The patient was discharged in stable condition after receiving appropriate post-procedural management and showed no further complications at 5 months.

**Lessons:**

SI-RT can cause severe thromboembolic complications despite the lack of conclusive evidence linking it to such events, and prophylactic anticoagulants are not routinely recommended. These considerations highlight the need for vigilant inpatient monitoring and thorough patient education at discharge to promptly address potential complications, as well as the importance of establishing guidelines for antiplatelet therapy in SI-RT patients without contraindications to minimize risks.

## 1. Introduction

Thrombocytosis, defined as a platelet count exceeding 450K/μL (450 × 10^9^/L in International System of Units),^[1]^ is most commonly a reactive process triggered by conditions such as chronic inflammation, iron deficiency, malignancy, infection, or trauma.^[2]^ Among these, splenectomy is a well-documented cause of reactive thrombocytosis (RT), with 75%–82% of patients experiencing postoperative RT, even in the absence of underlying myeloproliferative disorders (MPD).^[3]^ This platelet elevation can be conceptually linked to an increased risk of thromboembolic complications, including venous thromboembolism and arterial thrombotic events such as myocardial infarction (MI), pulmonary thromboembolism (PTE), and cerebrovascular accidents (CVA). Nevertheless, the direct causative relationship between splenectomy-induced RT (SI-RT) and these complications remains incompletely established.

We recently encountered a case in which a splenectomy was performed to treat a huge splenic hematoma in a patient without identifiable risk factors. Postoperatively, the patient developed an acute ST-segment elevation MI, a potentially fatal event that was successfully managed with prompt and appropriate intervention. In this report, therefore, we aim to discuss the management strategies for SI-RT, with a review of the relevant literature, to manage this rare but critical complication.

## 2. Case presentation

**Figure 1.**
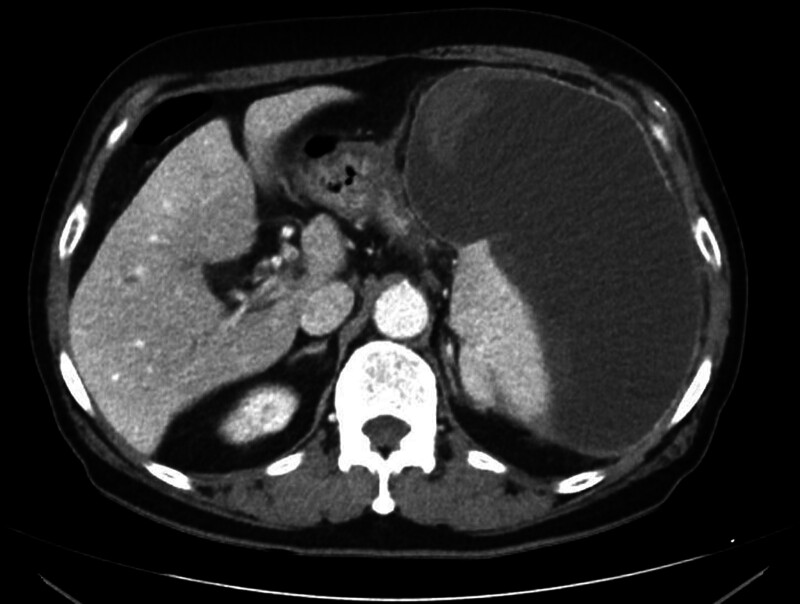
Contrast-enhanced computed tomography scan of the abdomen demonstrating a large splenic hematoma with well-defined margins, without evidence of active bleeding or surrounding hemoperitoneum.

**Figure 2.**
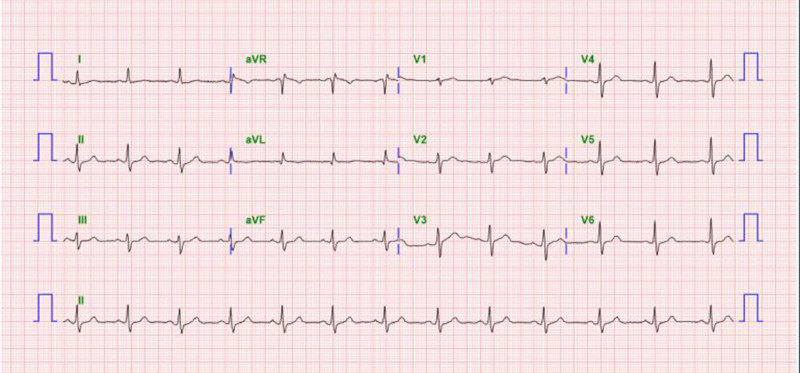
Standard 12-lead electrocardiogram showing normal sinus rhythm with no evidence of ischemic changes, arrhythmias, or conduction abnormalities.

**Figure 3.**
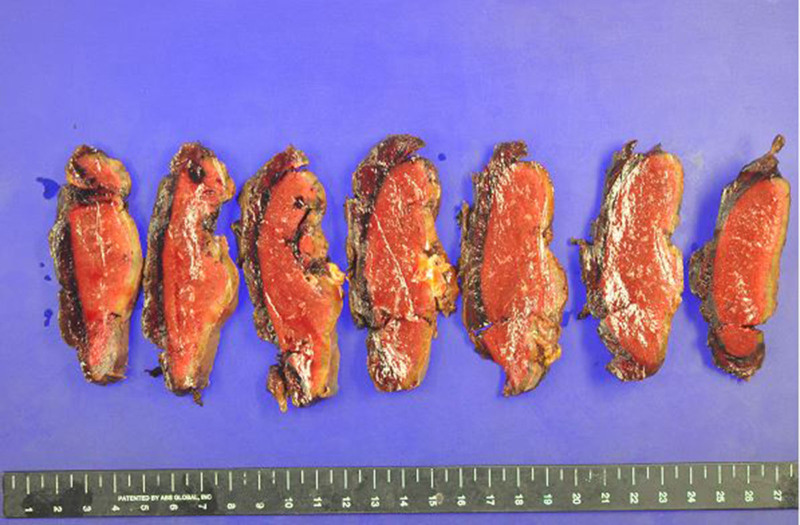
Gross pathological examination of the spleen, showing well-defined areas of hematoma without evidence of active hemorrhage or pathological abnormalities.

**Figure 4.**
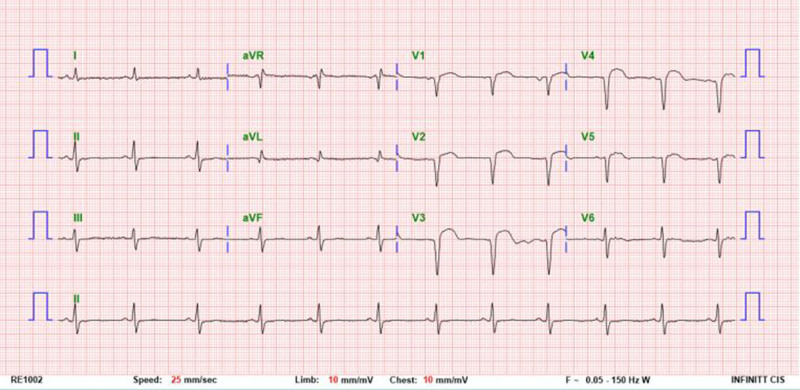
Standard 12-lead electrocardiogram showing prominent ST-segment elevation in leads V1, V2, and V3, indicative of acute anterior wall myocardial infarction.

**Figure 5.**
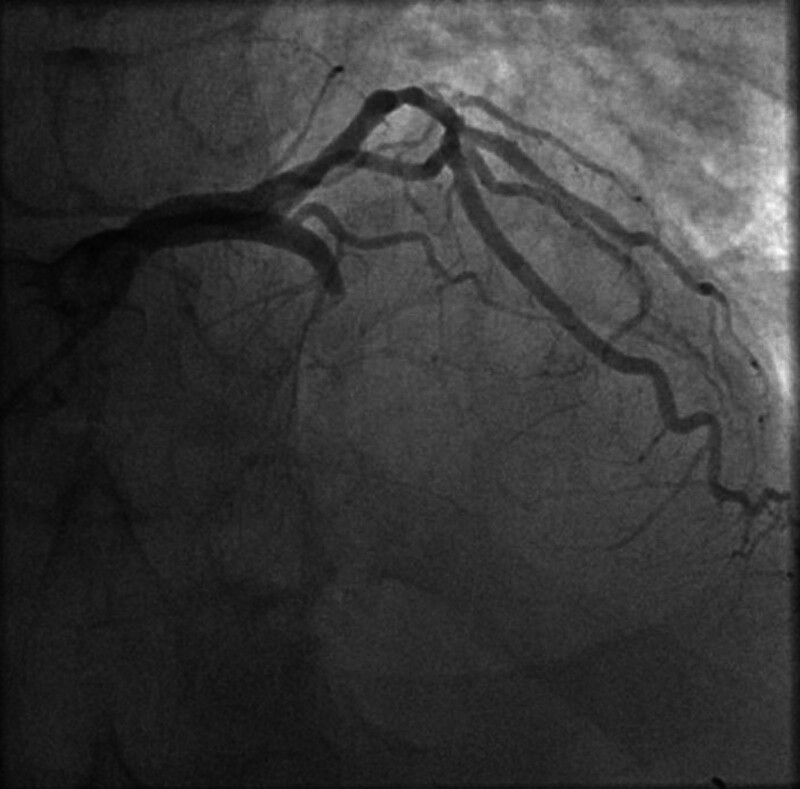
Coronary angiography showing complete occlusion of the proximal left anterior descending artery, with additional moderate stenoses observed in other coronary vessels.

**Figure 6.**
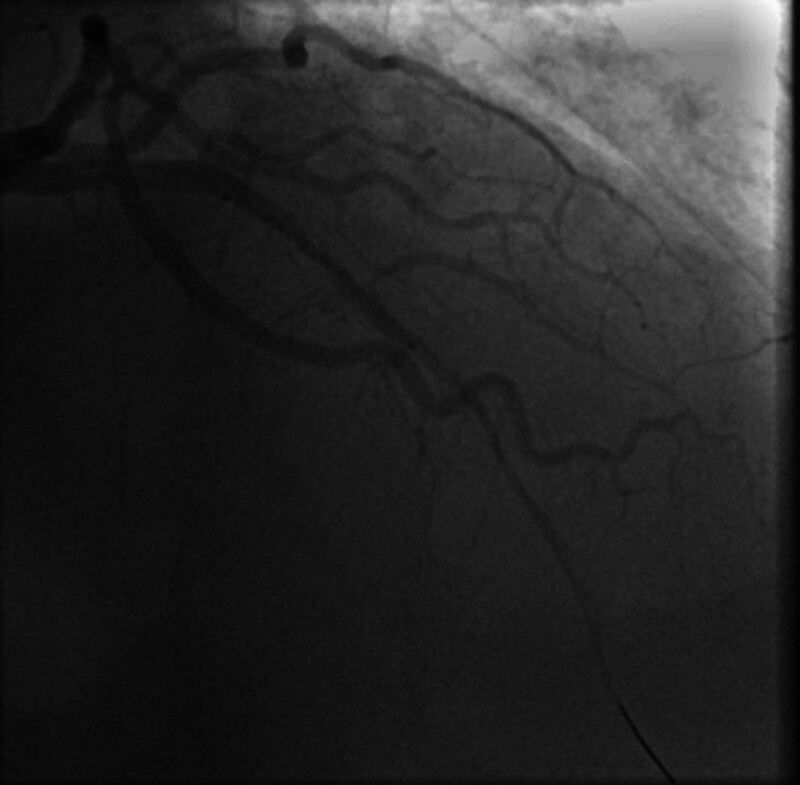
Coronary angiography after successful percutaneous coronary intervention, showing restored blood flow in the left anterior descending artery following the deployment of a drug-eluting stent.

This report was approved by the Institutional Review Board of Bucheon St. Mary’s Hospital, College of Medicine, The Catholic University of Korea (HC24ZISI0092), and the consent process was waived under the Institutional Review Board’s approval. A 72-year-old male presented to the emergency department with dyspnea as the primary symptom, and the patient also reported dyspepsia, fatigue, and dizziness. His medical history was notable only for hypertension, with no evidence of other significant comorbidities. On physical examination, tenderness and a palpable mass were identified in the left flank, raising suspicion of a surgical abdomen. Consequently, an urgent contrast-enhanced computed tomography scan was performed (Fig. 1), which revealed a large hematoma without signs of active rupture. There was no history of recent trauma, and the splenic hematoma was presumed to be spontaneous in nature. As a result, we planned to operate on this patient for the large symptomatic splenic hematoma; however, given the patient’s stable clinical condition, an emergency operation was deemed unnecessary. Instead, a plan was established to proceed with elective surgery following comprehensive preoperative evaluation. The patient demonstrated good functional status, and preoperative assessments, including electrocardiography and laboratory investigations, revealed no significant abnormalities, with a platelet count of 350K/μL (Fig. 2). Furthermore, a plain chest radiograph performed to investigate the cause of dyspnea showed no remarkable findings. On the 3rd day of hospitalization, an elective splenectomy was performed without intraoperative complications, and the histopathological examination confirmed the lesion to be a simple hematoma (Fig. 3). The postoperative course was uneventful. On postoperative day (POD) 7, routine laboratory tests performed as part of our usual practice revealed a platelet count of 541K/μL, which was interpreted as a nonspecific elevation following splenectomy. As the patient exhibited no specific clinical symptoms, no additional diagnostic evaluations were conducted. In line with our routine approach, prophylactic aspirin therapy (100 mg orally) was initiated, and serial follow-up of platelet counts was planned. On POD 11, the day scheduled for discharge, the patient unexpectedly developed sudden-onset severe epigastric pain accompanied by vomiting. Although abdominal evaluation was typically prioritized for such symptoms, the patient’s platelet count, measured at 972K/μL that morning, raised concerns about thrombotic complications. Therefore, empirical intravenous nitrates were promptly administered, and emergency coronary assessments were performed, revealing an elevated troponin T level of 767 pg/mL and electrocardiography findings of significant ST-segment elevation (Fig. 4). Coronary angiography was subsequently performed after consultation with cardiology, which revealed approximately 50% stenosis in the left circumflex artery and the right coronary artery, as well as total occlusion of the proximal left anterior descending artery, findings indicative of triple-vessel disease (Fig. 5). In light of these findings, a second-generation drug-eluting stent was successfully deployed in the proximal left anterior descending artery, while the remaining lesions were managed conservatively, followed by the initiation of standard post-procedural management, along with hydroxyurea for thrombocytosis (Fig. 6). The patient recovered well without any specific complications and was discharged on POD 15. At the 5-month follow-up, the patient’s general condition remained stable, with a left ventricular ejection fraction of 40% and a platelet count of 310K/μL.

## 3. Discussion

Evidence linking RT to complications such as MI, PTE, and CVA is limited.^[4]^ The largest study addressing this association was a retrospective analysis of 732 patients with platelet counts exceeding 500K/μL, conducted between 1991 and 1995.^[2]^ In this cohort, 12.3% were diagnosed with essential thrombocytosis (ET), while 87.7% had RT. ET was associated with higher platelet counts and a significantly increased frequency of both arterial and venous thrombotic complications. In contrast, RT was not significantly associated with thromboembolic events in the absence of other risk factors. Furthermore, a retrospective study conducted between 1984 and 1989 on 280 patients with platelet counts exceeding 1000K/μL, reported that 82% had RT, 14% had MPD, and 4% had an undetermined etiology.^[5]^ Notably, the incidence of thromboembolic complications in RT was reported to be only 4% in this study. This lack of evidence appears to be even more pronounced when specifically considering SI-RT. While several case reports have documented thromboembolic complications, including arterial thrombotic events, in patients with SI-RT, the majority of these cases involved individuals with underlying hematologic disorders.^[6,7]^ In contrast, the occurrence of MI in a previously healthy patient with no hematologic comorbidities, as presented in this case report, appears exceedingly rare, with only a single similar case reported in the literature.^[8]^

As previously discussed, we consider that the occurrence of SI-RT might be well-supported by existing evidence. However, the extent to which SI-RT progresses to complications, as well as the crucial question of what medical interventions should be employed during the SI-RT stage to prevent complications, remain significant areas of concern. In a retrospective study of 318 patients, excluding those with MPD or leukemia, SI-RT was observed in 75% of cases.^[9]^ Among these, 3.8% experienced thromboembolic complications, which was higher than the 1.3% observed in the control group with normal platelet counts; however, this difference did not demonstrate statistical significance. The study concluded that routine prophylactic use of anticoagulants or antiplatelet agents, such as aspirin, is not recommended based on these findings. Nonetheless, our perspective differs. Although thromboembolic complications related to SI-RT are rarely observed, which limits the establishment of robust evidence regarding their incidence, management, and prognosis, the possibility of their occurrence cannot be definitively excluded, and given their potentially critical nature, especially when they manifest as major complications such as MI, CVA, or PTE, careful consideration is warranted. Our patient exhibited overt symptoms on the scheduled day of discharge, which allowed for timely intervention, as SI-RT had already been identified and closely monitored based on preceding platelet trends. Although appropriate treatment was initiated without delay in this case, if any step in this process had been overlooked, the outcome could have been fatal. In our case, moreover, MI occurred despite prophylactic administration of low-dose aspirin (100 mg/d), raising the possibility of aspirin resistance or the inadequacy of single-agent prophylaxis in certain high-risk settings. This observation might suggest the need to consider whether more intensive antiplatelet strategies, such as dual therapy, might be warranted in selected patients, although current evidence remains insufficient.

## 4. Conclusion

In conclusion, given the favorable circumstances in this context, it would be prudent not to assume that such outcomes can consistently be anticipated. Thus, it seems necessary to establish further consensus on the use of antiplatelet therapy, such as aspirin, in patients with SI-RT, particularly in cases without specific contraindications. Furthermore, distinguishing between RT and ET following splenectomy is crucial, as ET carries a higher risk of complications compared to RT. Typically, SI-RT peaks within 1 to 3 weeks postoperatively and normalizes within a few months; any deviation from this pattern should prompt suspicion of ET.^[3]^ Finally, as demonstrated in our case, critical complications associated with SI-RT can occur even with prophylactic aspirin treatment. Therefore, it is important to ensure adequate inpatient observation and to educate patients at discharge to seek immediate medical attention in the event of symptom onset.

## Author contributions

**Conceptualization:** Chulhyo Jeon, Kiyoung Sung, Jinbeom Cho.

**Investigation:** Chulhyo Jeon, Kiyoung Sung.

**Methodology:** Chulhyo Jeon, Kiyoung Sung.

**Supervision:** Jinbeom Cho.

**Validation:** Chulhyo Jeon, Kiyoung Sung, Jinbeom Cho.

**Writing – review & editing:** Chulhyo Jeon, Kiyoung Sung, Jinbeom Cho.

**Writing – original draft:** Jinbeom Cho.
